# Filling gaps on ivermectin knowledge: effects on the survival and reproduction of *Anopheles aquasalis*, a Latin American malaria vector

**DOI:** 10.1186/s12936-016-1540-y

**Published:** 2016-09-22

**Authors:** Vanderson S. Sampaio, Tatiana P. Beltrán, Kevin C. Kobylinski, Gisely C. Melo, José B. P. Lima, Sara G. M. Silva, Íria C. Rodriguez, Henrique Silveira, Maria G. V. B. Guerra, Quique Bassat, Paulo F. P. Pimenta, Marcus V. G. Lacerda, Wuelton M. Monteiro

**Affiliations:** 1 Diretoria de Ensino e Pesquisa, Fundação de Medicina Tropical Dr. Heitor Vieira Dourado, Manaus, Brazil; 2 Escola Superior de Ciências da Saúde, Universidade do Estado do Amazonas, Manaus, Brazil; 3 Sala de Análise de Situação em Saúde, Fundação de Vigilância em Saúde do Amazonas, Manaus, Brazil; 4 Armed Forces Research Institute of Medical Sciences, Bangkok, Thailand; 5 Instituto Oswaldo Cruz, FIOCRUZ, Rio de Janeiro, Brazil; 6 Instituto de Higiene e Medicina Tropical, Universidade Nova de Lisboa, Lisbon, Portugal; 7 ISGlobal, Barcelona Ctr. Int. Health Res. (CRESIB), Hospital Clínic-Universitat de Barcelona, Barcelona, Spain; 8 Centro de Investigação em Saúde de Manhiça (CISM), Maputo, Mozambique; 9 Centro de Pesquisas René Rachou, FIOCRUZ, Belo Horizonte, Brazil; 10 Instituto de Pesquisas Leônidas & Maria Deane, FIOCRUZ, Manaus, Brazil

**Keywords:** Malaria elimination, Vector control, Ivermectin, *Anopheles aquasalis*, Amazon

## Abstract

**Background:**

Strategies designed to advance towards malaria elimination rely on the detection and treatment of infections, rather than fever, and the interruption of malaria transmission between mosquitoes and humans. Mass drug administration with anti-malarials directed at eliminating parasites in blood, either to entire populations or targeting only those with malaria infections, are considered useful strategies to progress towards malaria elimination, but may be insufficient if applied on their own. These strategies assume a closer contact with populations, so incorporating a vector control intervention tool to those approaches could significantly enhance their efficacy. Ivermectin, an endectocide drug efficacious against a range of *Anopheles* species, could be added to other drug-based interventions. Interestingly, ivermectin could also be useful to target outdoor feeding and resting vectors, something not possible with current vector control tools, such as impregnated bed nets or indoor residual spraying (IRS).

**Results:**

*Anopheles aquasalis* susceptibility to ivermectin was assessed. In vivo assessments were performed in six volunteers, being three men and three women. The effect of ivermectin on reproductive fitness and mosquito survivorship using membrane feeding assay (MFA) and direct feeding assay (DFA) was assessed and compared. The ivermectin lethal concentration (LC) values were LC_50_ = 47.03 ng/ml [44.68–49.40], LC_25_ = 31.92 ng/ml [28.60–34.57] and LC_5_ = 18.28 ng/ml [14.51–21.45]. Ivermectin significantly reduced the survivorship of *An. aquasalis* blood-fed 4 h post-ingestion (*X*^*2*^ [N = 880] = 328.16, p < 0.001), 2 days post-ingestion (DPI 2) (*X*^*2*^ [N = 983] = 156.75, p < 0.001), DPI 7 (*X*^*2*^ [N = 935] = 31.17, p < 0.001) and DPI 14 (*X*^*2*^ [N = 898] = 38.63, p < 0.001) compared to the blood fed on the untreated control. The average number of oviposited eggs per female was significantly lower in LC_5_ group (22.44 [SD = 3.38]) than in control (34.70 [SD = 12.09]) (*X*^*2*^ [N = 199] = 10.52, p < 0.001) as well as the egg hatch rate (LC_5_ = 74.76 [SD = 5.48]) (Control = 81.91 [SD = 5.92]) (*X*^*2*^ [N = 124] = 64.24, p < 0.001). However, no differences were observed on the number of pupae that developed from larvae (Control = 34.19 [SD = 10.42) and group (LC_5_ = 33.33 [SD = 11.97]) (*X*^*2*^ [N = 124] = 0.96, p > 0.05).

**Conclusions:**

Ivermectin drug reduces mosquito survivorship when blood fed on volunteer blood from 4 h to 14 days post-ingestion controlling for volunteers’ gender. Ivermectin at mosquito sub-lethal concentrations (LC_5_) reduces fecundity and egg hatch rate but not the number of pupae that developed from larvae. DFA had significantly higher effects on mosquito survival compared to MFA. The findings are presented and discussed through the prism of malaria elimination in the Amazon region.

## Background

Malaria remains an important public health problem worldwide affecting mainly underdeveloped and developing countries in Africa, Asia and Latin America. The World Health Organization (WHO) estimated that 214 million cases of malaria occurred worldwide in 2015 [1]. Malaria elimination and eradication are present themes on WHO’s agenda for infectious diseases [2, 3]. Research institutes and policy makers have made great efforts worldwide in order to achieve significant reduction in malaria incidences, with the ambitious long-term aim of global eradication [4–7]. Approaches designed to progress towards malaria elimination must rely on the detection and treatment of infections, rather than fever, and comprise the concomitant use of different tools concerning health surveillance improvement through technologies, applying transmission blocking by development of vaccines, high sensibility new generation rapid tests, insecticides and drugs that can, among other features, circumvent the resistance issue [8].

Strategies focused on mass screening and treatment (MSAT) and variations of it, such as focused screening and treatment (FSAT) and reactive case detection (RCD), sometimes are described as success cases, but these strategies depend on several factors that can drive for failure, such as logistics, public health policies, population coverage, and even diagnostic tool sensitivity [9]. Likewise, mass drug administration (MDA) using artemisinin-based combination therapy (ACT) has been shown to be an effective strategy, as well as MSAT, for high-incidence scenarios. However, issues like community acceptance and drug resistance increasing are still relevant concerns [9]. These are potential control measures that can be improved by integration with effective vector control interventions. Extensive use of long lasting impregnated nets and indoor residual spray has led to a change in the vector comportment from indoor to outdoor feeding and resting behaviour [10, 11]. This shift brings a new challenge to target outdoor malaria transmission in a sustainable way in order to achieve elimination [12].

Ivermectin has proven to be effective against a range of *Anopheles* species [13–16]. Ivermectin can impact four of five vectorial capacity variables, including daily probability of adult mosquito survivorship, daily probability a mosquito feeds on a human, vector competence, and vector density in relation to the host [17–20]. Treating hosts with a systemic insecticide, such as ivermectin, could circumvent the issue of outdoor transmission, as it would target the vector regardless of feeding habit location and time [17]. In addition of having an excellent safety profile in humans, ivermectin has proven to be effective against a range of other neglected diseases, such as filariasis and helminthiasis [21]. Furthermore, the drug presents features in agreement with some of the malaria eradication research agenda (malERA) initiative recommendations, such as reducing adult mosquito survival rates, shifting age structure, reducing the proportion of older females, and targeting outdoor feeding and resting [6]. Moreover, if livestock are treated with ivermectin for malaria control, then this is coherent with the *One Health* concept since it acts against livestock parasites, improving both economic output and nutrient availability [22].

Ivermectin MDA, even when a single round is applied, reduces the survivorship of mosquitoes, shifts the mosquito population age structure, and decreases sporozoite rate [23]. Modelling suggests that adding ivermectin as an adjunct during ACT MDA could reduce malaria transmission and significantly reduce the number of MDAs and time to elimination [24]. Ivermectin has been used in MDA in Latin America for onchocerciasis control [25] and this infection has been eliminated in four of the six endemic countries. This illustrates that ivermectin MDA can be effectively implemented in Latin America for disease elimination. Indigenous populations are currently under ivermectin MDA intervention for onchocerciasis control in the Brazil-Venezuela border [26]. Variations in the mosquitocidal effect between anopheline species [27] and blood meals [28] make essential local studies regarding these features that directly affect the timing of ivermectin administration, a crucial parameter to form a useful addition to anti-malarial drugs [29].

*Anopheles aquasalis* seems to play an important role in malaria transmission in coastal regions of Latin America. Infection rates due to *Plasmodium vivax* were previously reported ranging from 0.5 to 1.7 %, both in outdoor and indoor resting mosquitoes in Venezuela [30], and in Brazil the infection rate was estimated 1.18 % [31]. Since mosquito colonies have been established, the species has been used as a model for assessing vector-parasite interactions [32]. *Anopheles aquasalis* has been described as presenting variable feeding behaviour, both anthropophilic and zoophilic [30, 31]. It was also designated as a widely distributed and abundant species [32, 33], being reported both at Atlantic and Pacific coasts, from Central America to southern Brazil [32]. It has been demonstrated that the species has both indoor and outdoor feeding and resting behaviour as well [27, 34, 35]. Furthermore, *An. aquasalis* has been described as zoophilic species in Amazon region [30, 36]. Such features allow to classify the species as of great importance for the Latin America.

Even though much evidence has been generated regarding ivermectin effects on malaria transmission, some questions remain unanswered regarding its effects on the vector’s biology [21, 37]. Here the ivermectin effects on the survivorship and reproductive fitness of the American malaria vector *An. aquasalis* were assessed. The differences of ivermectin effect on mosquito survivorship using membrane feeding assay (MFA) and direct feeding assay (DFA) from drug-treated volunteers were also evaluated.

## Methods

### Mosquito colony

*Anopheles aquasalis* specimens were obtained from a well-established colony at the Entomology Department Insectary of the *Fundação de Medicina Tropical Dr Heitor Vieira Dourado* (FMT-HVD). Mosquitoes were raised at 26–27 °C, 70–80 % relative humidity and 12/12 light/dark photoperiod. Larvae were fed on commercial fish food (Tetramin Gold^®^) and adults were provided ad libitum with 10 % sucrose solution. Three to five days post emergence female mosquitos were used in all experiments.

### Experimental drugs

Ivermectin tablets (Abbot Laboratórios do Brasil©) were supplied by FMT-HVD and the dosage was fitted according to volunteer weight in order to have a final dosage of 200 µg/kg body weight, in agreement with dosages used during onchocerciasis MDA. Tablets of 6 mg were given according to weight band (51–65 kg = 2 tablets; 66–79 kg = 2 ½ tablets; and >80 kg = 3 tablets) following the dosage recommendations. Powdered ivermectin compound was obtained from Sigma-Aldrich (St Louis, MO, USA) for the estimation of LC_50_ and reproductive fitness assays.

### Volunteer enrolment

Subjects of both genders with medical recommendations on the use of ivermectin, according to the National Health Surveillance Agency (ANVISA), were enrolled for two assays: mosquito survivorship and blood-feeding type comparison, each with three male and three female volunteers.

For LC_50_ estimates and reproductive fitness experiments, a single volunteer was enrolled for each objective. Volunteers under any treatment for diseases other than those mentioned, pregnant, under 18 years old, or planning to travel were not enrolled.

### In vitro LC_50_ estimates

Powdered ivermectin compound was dissolved in dimethylsulfoxide to 10 mg/ml and aliquots were frozen at −20 °C. Before each experiment, ivermectin aliquots were diluted in phosphate buffered saline (PBS) and 10 µl of different concentrations of drug were added to 990 µl of blood to achieve the final concentration for blood-fed mosquitoes as described in detail elsewhere [20]. Blood samples from a single untreated volunteer were used as control in all experiments.

Blood meal was kept at 36 °C throughout the MFA, which lasted 30 min. Approximately 70 mosquitoes per treatment group were offered blood meal in order to have at least 50 engorged specimens. Fully engorged mosquitoes were gently transferred to 500-ml cardboard containers and kept under the same conditions as described above for the colonized mosquitoes. Every 24 h dead mosquitoes were removed and counted until the fifth day. Five experimental replicates of each ivermectin concentration were performed in order to estimate the lethal concentrations in 5 days.

### Effects of ivermectin drug treatment on mosquito survivorship

Three male and three non-pregnant female volunteers were enrolled in pairs for this experiment. Five ml of blood samples were collected at specific time points: (i) before drug ingestion (BDI); (ii) 4 h post-ingestion (HPI 4); (iii) 2 days post-ingestion (DPI 2); (iv) 4 days post-ingestion (DPI 4); (v) 7 days post-ingestion (DPI 7); and, (vi) 14 days post-ingestion (DPI 14). The BDI samples served as baseline control. Blood samples were maintained at 36 °C for MFA. Approximately 70 mosquitoes were blood fed during 30 min in order to have at least 50 fully engorged specimens. Engorged females were gently transferred to a 500-ml cardboard container and kept at same conditions described for LC_50_ calculations. Dead mosquitoes were removed daily for 10 days and data were recorded. Mosquitoes fed in blood collected BDI were used as controls. No parallel controls were used.

### Effects on reproductive fitness

Approximately 100 *An. aquasalis* specimens were submitted to three replicates for MFA with blood meals containing a sub-lethal concentration of ivermectin (LC_5_). Ten fully engorged female mosquitoes were gently transferred to a cage containing a water bowl surrounded with a moist filter paper for oviposition. They were provided ad libitum with 10 % sucrose solution. After 3 days, gravid females were dissected in order to identify retained eggs. The number of eggs laid per female (fecundity), number of eggs producing larvae (egg hatch rate) and number of pupae that developed from larvae, were counted on the third, fifth and seventh days post-blood meal. Eggs, larvae and pupae were transferred to new containers after each counting in order to wait for the next instar.

### Comparison of mosquito survivorship from MFA and DFA

Four experimental replicates were performed with three male and three female volunteers divided in two experimental groups with 60–70 mosquitoes for the DFA and MFA. Four hours post drug ingestion, a 5-ml blood sample was collected from the volunteer for MFA and immediately offered to mosquitoes. Simultaneously, a DFA was performed in the same volunteer for 30 min. Then, fully engorged females were gently transferred to 0.5-l containers for mortality observation as described above. Ten freshly engorged mosquitoes from each experimental group were quickly cold anesthetized at −20 °C and weighed. In order to exclude the blood meal volume ingested as a confounder, their weights were compared. Blood-fed mosquitoes were monitored daily and had mortality data annotated as mentioned above until the last specimen died.

### Data analysis

A non-linear mixed model with probit analysis was applied to estimate in vitro LC_50_, LC_25_ and LC_5_ values. Lethal concentration experiments with mortality background greater than 20 % were discarded and control mortality background lower than 20 % was corrected by the Abbot formula [39].

Kaplan–Meier survival analysis followed by Mantel-Cox Log-rank test was used to evaluate both the drug effects on the survivorship of mosquitoes and differences between MFA and DFA. Additionally, proportional hazard ratio was estimated by shared frailty Cox regression models using Breslow method in view of controlling for volunteer gender and multiple observations from the same volunteer on the survival analysis.

Differences between control and LC_5_ samples regarding ivermectin effects on number of eggs laid per female (fecundity), number of eggs that produced larvae (egg hatch rate) and number of pupae that developed from larvae were estimated by a non-parametric equality-of-medians test once the sample was not assumed to be normal distributed by the Shapiro–Wilk test.

All data was double entered in spreadsheets and Stata software v13 (StataCorp. 2013. Stata Statistical Software: Release 13. College Station, TX: StataCorp LP) was used for the analyses.

## Results

### LC_50_ estimation

Lethal concentrations were estimated according to data described in Table 1. LC_50_ fed to *An. aquasalis* was estimated as LC_50_ = 47.03 ng/ml [95 % CI 44.68–49.40], LC_25_ = 31.92 ng/ml [95 % CI 28.60–34.57] and LC_5_ = 18.28 ng/ml [95 % CI 14.51–21.45] (n = 1415–5 experimental replicates) (Table 1).

**Table 1:** Lethal concentrations of ivermectin for *Anopheles aquasalis*

LC (%)	Drug concentration (ng/ml) [95 % CI]
5	18.28 [14.51–21.45]
10	22.52 [18.73–25.62]
15	25.92 [22.23–28.90]
20	29.00 [25.47–31.81]
25	31.92 [28.60–34.57]
30	34.79 [31.70–37.29]
40	40.66 [38.03–42.91]
50	47.03 [44.68–49.40]

*LC* lethal concentration

### Effects on the mosquito survivorship

**Fig. 1:**
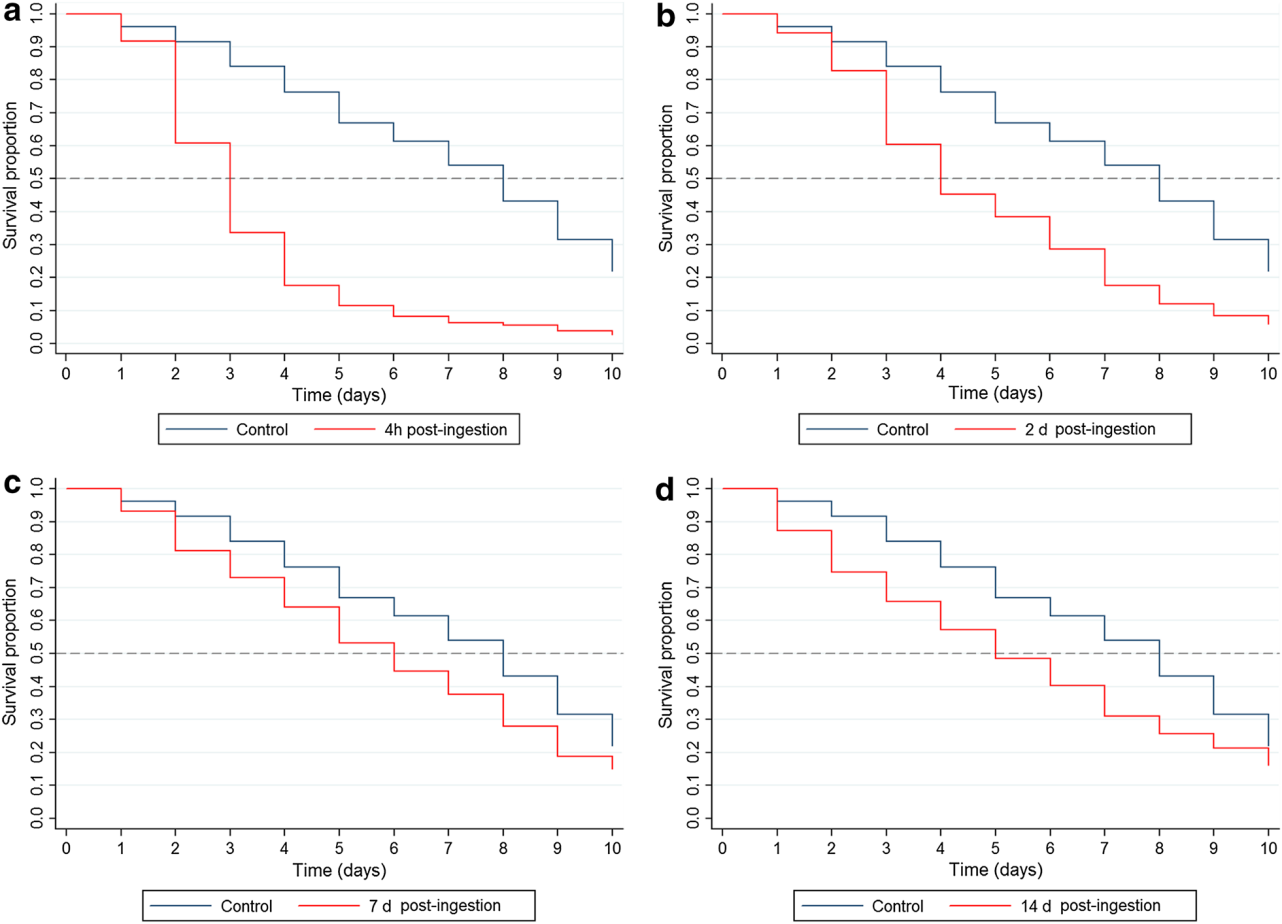
Effects of ivermectin on the survivorship of *Anopheles aquasalis.* **a** Mosquitoes fed on a volunteer blood meal with ivermectin 4 h post ingestion (HPI 4); **b** Mosquitoes fed on volunteers’ blood meal with ivermectin 2 days post ingestion (DPI 2); **c** Mosquitoes fed on volunteers’ blood meal with ivermectin 7 days post ingestion (DPI 7); **d** Mosquitoes fed on volunteers’ blood meal with ivermectin 14 days post ingestion (DPI 14)

*Anopheles aquasalis* had significantly reduced survivorship when blood fed on volunteer blood containing ivermectin HPI 4 (*X*^*2*^ [N = 880] = 328.16, p < 0.001), DPI 2 (*X*^*2*^ [N = 983] = 156.75, p < 0.001), DPI 7 (*X*^*2*^ [N = 935] = 31.17, p < 0.001) and DPI 14 (*X*^*2*^ [N = 898] = 38.63, p < 0.001) compared to the blood fed on the untreated control. While it took approximately 6 days to have 50 % of the mosquitoes dead in DPI 14, this time decreases to 4 and 3 days in DPI 2 and HPI 4, respectively (Fig. 1). Regression model revealed a dose–response effect on hazard ratios (HR) for time post-ingestion (TPI). The HR increases while the TPI decreases. Proportion of dead mosquitoes was threefold increased for mosquitoes submitted to ivermectin blood meals HPI 4, and 44 % higher in mosquitoes offered to ivermectin blood meals DPI 14 (Table 2).

**Table 2:** Shared frailty Cox model of time post-ingestion effects on *Anopheles aquasalis* survivorship

	HR [95 % CI]	p value
Time post ingestion		
Control	1	–
HPI 4	3.184 [2.775–3.653]	0.0001
DPI 2	1.972 [1.734–2.244]	0.0001
DPI 5	1.727 [1.510–1.976]	0.0001
DPI 7	1.380 [1.213–1.572]	0.0001
DPI 14	1.437 [1.259–1.640]	0.0001

Hazard ratios for time post-ingestion

*HPI* hours post ingestion, *DPI* days post ingestion, *LR* likelihood-ratio

### Effects on reproductive fitness

**Fig. 2:**
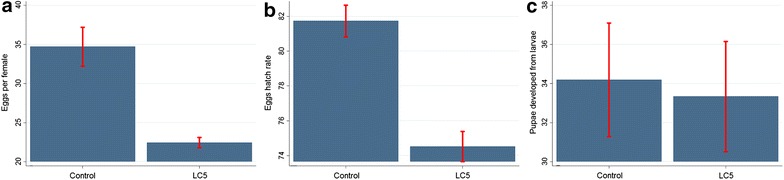
Effects of ivermectin on the reproductive fitness of *Anopheles aquasalis.* **a** Effects on number of eggs per female (fecundity); **b** Effects on eggs that produced larvae (eggs hatch rate); **c** Effects on number of pupae that developed from larvae

Reproductive fitness was affected when mosquitoes were submitted to a 5 % lethal concentration (LC_5_) (18.28 ng/ml [95 % CI 14.51–21.45]). A total of 199 blood-fed mosquitoes were allowed to egg laying substrate. In the control group, average number of oviposited eggs per female (34.70 [SD = 12.09]) was significantly higher than in LC_5_ group (22.44 [SD = 3.38]) (Fig. 2a) (*X*^*2*^ [N = 199] = 10.52, p < 0.001). The average number of hatched eggs that produced larvae (egg hatch rate) was also significantly higher in control (81.91 [SD = 5.92]) than in LC_5_ group (74.76 [SD = 5.48]) (Fig. 2b) (*X*^*2*^ [N = 124] = 64.24, p < 0.001). Regarding the number of pupae that developed from larvae, no differences were observed between the control (34.19 [SD = 10.42) and LC_5_ group (33.33 [SD = 11.97]) (Fig. 2c) (*X*^*2*^ [N = 124] = 0.96, p > 0.05).

### Comparison between MFA and DFA

**Fig. 3:**
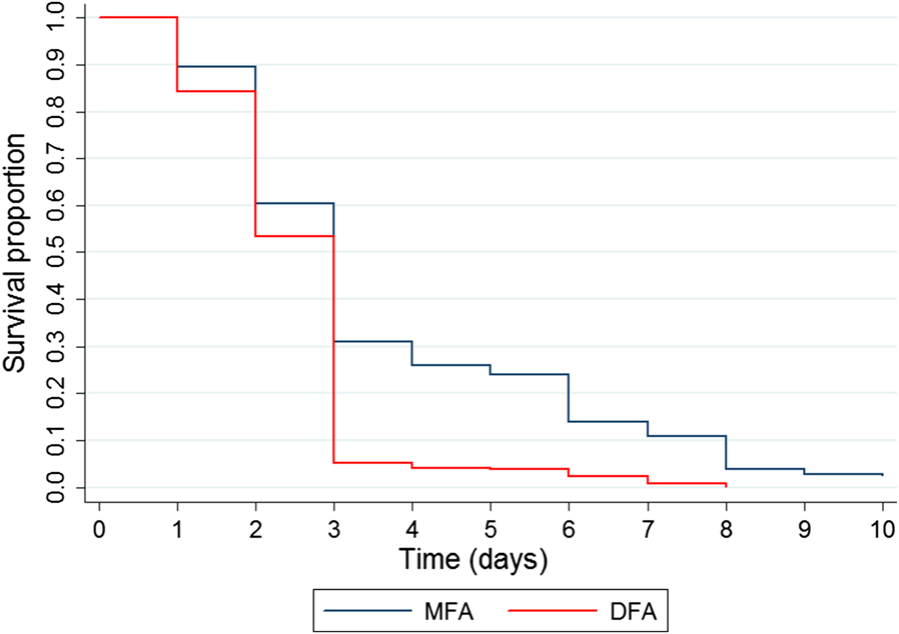
Kaplan–Meier survival function curves. Comparison of different blood meal types. Survival proportion significantly increased in DFA compared with MFA (*X* ^*2*^ [N = 2.623] = 0.2, p < 0.05). *MFA* membrane feeding assay, *DFA* direct feeding assay

**Fig. 4:**
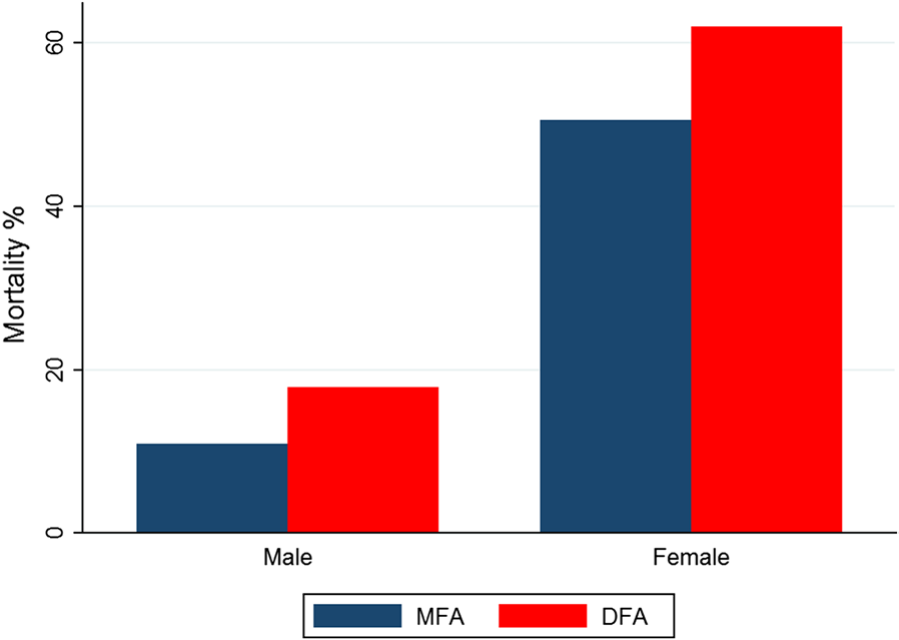
Mortality proportion of mosquitoes fed with blood containing ivermectin at the second day after blood meals. Comparison of MFA and DFA methods (p < 0.001) and between male and female volunteers (p < 0.001). *MFA* membrane feeding assay, *DFA* direct feeding assay

A total of 2639 fully engorged females were obtained from the blood-feeding assays, being 777 (29.44 %) subjected to MFA and 1862 (70.56 %) from the DFA. There were no significant differences between blood-fed mosquito weight from DFA (0.040 mg [SD = 0.02]) or MFA (0.059 mg [SD = 0.02]) experimental groups (t = 1.52 [p > 0.05]). Survivorship of *An. aquasalis* blood fed in DFA was significantly reduced compared to MFA (Fig. 3) (*X*^*2*^ [N = 2.623] = 147.48, p < 0.001). Mosquitoes blood fed by DFA died faster than MFA. At the third day after blood meals, the survival proportion of *An. aquasalis* was less than 10 % at day 3 for DFA while it was 30 % for MFA (Fig. 3). Mortality percentage 2 days after feeding assays was significantly higher both in DFA compared to MFA (*X*^*2*^ [N = 2.623] = 0.2, p < 0.05) and female compared to male volunteers (*X*^*2*^ [N = 2.623] = 412.7, p < 0.001) (Fig. 4).

Shared frailty Cox model showed that DFA blood-fed mosquitoes compared to MFA had a 73 % increase of mortality rate adjusting for volunteers’ gender (HR = 1.726 [1.573–1.895] p = 0.0001). Once more, volunteers’ gender was assessed as an effect modifier and the regression model revealed an increase of risk for women volunteers (1.409 [1.295–1.532] p < 0.001) (Table 3).

**Table 3:** Shared frailty Cox model of feeding assay effect on *Anopheles aquasalis* survivorship

	HR [95 % CI]	p value
Feeding type		
MFA	1	–
DFA	1.726 [1.573–1.895]	0.0001
Gender		
Male	1	–
Female	1.314 [1.199–1.442]	0.0001

*MFA* membrane feeding assay, *DFA* direct feeding assay

## Discussion

Malaria elimination is an ambitious objective that has now been seriously considered and embraced both by the public health community and scientists worldwide. In this scenario, ivermectin has appeared as a potential complementary tool for elimination as it effectively targets outdoor transmission, has a novel mechanism of action that might bypass occurrence of resistance and could utilize implementation mechanisms that are already functional because of efforts to control other diseases, such as onchocerciasis and lymphatic filariasis [21]. Moreover, the drug has been reported to reduce vectorial capacity for *Plasmodium* transmission, both by reducing mosquito survival and possibly inhibiting *Plasmodium falciparum* sporogony [38, 39]. Even so, and despite recent discoveries, little is known about the effects of the drug on the biology of different vectors, especially from Latin America [27].

In this study, the effects of ivermectin on *An. aquasalis* survivorship and reproduction are showed for the first time. Ivermectin was shown to increase mortality and reduce reproductive capacity of *An. aquasalis*. The *An. aquasalis* ivermectin lethal concentrations (LC_50_ = 47.03 ng/ml, LC_25_ = 31.92 ng/ml, LC_5_ = 18.28 ng/ml) are higher than calculated previously for *Anopheles gambiae* [17, 20] but still within human relevant range following oral treatment with 150–200 μg/kg [39, 40]. It must be noted that the methods used here and firstly used by Kobilinsky et al. [17] for LC estimates differ from others since an in vitro mixing of drug and blood was used instead of blood from treated subjects and this method could be influencing the higher LC values found here. Because single doses of 200 μg/kg can only keep blood concentrations compatible with this lethal concentrations for a short period, using higher or repeated doses or slow release formulations of ivermectin should be considered as a feasible strategy. These data allow to infer that ivermectin treatment of humans should impart a lethal effect on *An. aquasalis*.

In vivo data revealed that mosquitoes fed on volunteer blood containing ivermectin (200 µg/kg) at 4 h, 2, 4, 7, and 14 days post drug ingestion significantly reduced survivorship compared to those fed on untreated control individual blood. These findings are similar to Foley et al. [15] which showed survivorship reduction for *Anopheles farauti* until 14 days post ivermectin ingestion (250 μg/kg) by DFA. Ivermectin seems to have great affinity for adipose tissue. Strongly lipid binding may cause its slow release, thereby increasing its persistence in the body, as suggested previously [41, 42, 43]. This phenomenon may explain why mosquito lethal effects were observed as late as 14 days post drug ingestion. Increasing the dose of ivermectin would likely impart a greater effect against *An. aquasalis* for a longer period of time.

Ivermectin sub-lethal effects on the reproductive fitness of *Anopheles* mosquitoes were first reported by Gardner et al. [44] in *Anopheles quadrimaculatus* specimens fed canine blood containing ivermectin. Two studies indicate that ivermectin treatment of cattle reduces mosquito fecundity for *Anopheles coluzzii* [45] and *An. gambiae* s.s. [46]. A complete inhibition of *An. gambiae* fecundity when mosquitoes fed on human blood 24 h post treatment with a 150–200 µg/kg dosage was shown by Derua et al. [47]. The findings support and extend studies since was demonstrated that ivermectin effects on eggs/female proportion, eggs hatchability and even on pupae/larvae proportion under a low concentration dosage. Additionally it should be appreciated that human pharmacokinetic may differ from those in animals, as in the first three studies, domesticated animals were injected with doses varying from 6 to 600 µg/kg. These findings reinforce the hypothesis that even sub-lethal doses of ivermectin could play an important role on altering the vectorial capacity.

Studies conducted on ivermectin effects over mosquitoes are usually carried out through MFA [19, 42, 44]. As described previously, since ivermectin is lipophilic, it usually binds to fatty tissue where it may lead to higher concentrations in different compartments. This feature, in turn, led to believe that mosquitoes fed by DFA on sub-dermal capillaries may ingest higher ivermectin concentrations than mosquitoes fed by MFA with venous blood, imparting a greater mosquito lethal effect, as suggested by Chaccour et al. [27]. Here was also showed significant differences between MFA and DFA HRs (1.54 [1.406–1.684] p < 0.001) adjusting for volunteer gender. Although the limited number of volunteers (3 males and 3 females) may be a limitation for the study, these are exciting findings since previous results obtained from MFA studies may be underestimates of the real effects that occur during direct feeding after ivermectin MDA during a malaria elimination campaign. Regression model also revealed an increased risk for mosquitoes feeding on women volunteers independent of the blood feeding assay in accordance with a recent study reporting a greater availability of ivermectin in female human and in higher body mass indices volunteers [42]. Since only one single time point (4 h post-ingestion) was evaluated, additional studies must be carried out in order to assess these differences in later time-points where the effect of ivermectin decreases.

## Conclusions

Ivermectin has proven to be effective against a range of malaria vectors worldwide. The drug affects many aspects of both vector biology and its vectorial capacity as well. Considering the diversity of environment in the Amazon region and consequently of entomological and epidemiological scenarios, malaria elimination campaigns in Amazon must resort to concomitant multiple strategies. Here a gap of knowledge regarding ivermectin effects on an important Amazon vector species, *An. aquasalis* was filled. It was demonstrated that ivermectin impacts mosquito survivorship for up to 14 days post-ingestion and has a deleterious effect on the vector reproductive fitness. Significant difference between MFA and DFA was found and no difference concerning blood meal volume comparing MFA and DFA was shown. Considering the findings, malaria elimination strategies in the Amazon could benefit from having ivermectin as an additional tool, which would readily complement the effect of the use of drugs for population treatment, or other vector control mechanism. Since outdoor transmission in Amazon has a relevant contribution to the overall malaria transmission and the ivermectin way of action influences this, the drug would likely have an impact on the incidence of disease in the region. Furthermore, since *An. aquasalis* is incriminated both as zoophilic and anthropophilic, has a widespread distribution and is implicated in malaria transmission as well, it seems to be feasible the deployment of strategies focused on cattle and/or human treatment. Future investigation concerning ivermectin effects on other important Amazonian species, such as *Anopheles darlingi* and *Anopheles albitarsis*, should be assessed prior to widespread adoption of ivermectin as a malaria elimination tool in the Amazon.

## Abbreviations

MDA: mass drug administration
MSAT: mass screening and treatment
FSAT: focused screening and treatment
MFA: membrane feeding assay
DFA: direct feeding assay
LC: lethal concentration
WHO: World Health Organization
RCD: reactive case detection
ACT: artemisinin-based combination therapy
malERA: malaria eradication research agenda
ELISA: enzyme-linked immunosorbent assay
FMT-HVD: *Fundação de Medicina Tropical Dr. Heitor Vieira Dourado*
PBS: phosphate buffered saline
BDI: before drug ingestion
HPI 4: 4 h post ingestion
DPI 2: 2 days post ingestion
DPI 4: 4 days post ingestion
DPI 7: 7 days post ingestion
DPI 14: 14 days post ingestion
TPI: time post ingestion
HR: hazard ratios
